# In *Staphylococcus aureus* the regulation of pyruvate kinase activity by serine/threonine protein kinase favors biofilm formation

**DOI:** 10.1007/s13205-014-0248-3

**Published:** 2014-09-12

**Authors:** D. Vasu, M. M. Sunitha, L. Srikanth, V. Swarupa, U. Venkateswara Prasad, K. Sireesha, S. Yeswanth, P. Santhosh Kumar, K. Venkatesh, Abhijit Chaudhary, P. V. G. K. Sarma

**Affiliations:** 1 Department of Biotechnology, Sri Venkateswara Institute of Medical Sciences, Tirupati, AP 517507 India; 2 Department of Microbiology, Sri Venkateswara Institute of Medical Sciences, Tirupati, AP 517507 India

**Keywords:** *K*_M_, PK, Stpks, PknB, Biofilm

## Abstract

*Staphylococcus aureus,* a natural inhabitant of nasopharyngeal tract, survives mainly as biofilms. Previously we have observed that *S. aureus* ATCC 12600 grown under anaerobic conditions exhibited high rate of biofilm formation and l-lactate dehydrogenase activity. Thus, the concentration of pyruvate plays a critical role in *S. aureus*, which is primarily catalyzed by pyruvate kinase (PK). Analyses of the PK gene sequence (JN645815) revealed presence of PknB site in PK gene indicating that phosphorylation may be influencing the functioning of PK. To establish this hypothesis the pure enzymes of *S. aureus* ATCC 12600 were obtained by expressing these genes in PK 1 and PV 1 (JN695616) clones and passing the cytosolic fractions through nickel metal chelate column. The molecular weights of pure recombinant PK and PknB are 63 and 73 kDa, respectively. The enzyme kinetics of pure PK showed *K*_M_ of 0.69 ± 0.02 µM, while the *K*_M_ of PknB for stpks (stpks = NLCNIPCSALLSSDITASVNCAK) substrate was 0.720 ± 0.08 mM and 0.380 ± 0.07 mM for autophosphorylation. The phosphorylated PK exhibited 40 % reduced activity (PK = 0.2 ± 0.015 μM NADH/min/ml to P-PK = 0.12 ± 0.01 μM NADH/min/ml). Elevated synthesis of pyruvate kinase was observed in *S. aureus* ATCC 12600 grown in anaerobic conditions suggesting that the formed pyruvate is more utilized in the synthesis phase, supporting increased rate of biofilm formation.

**Electronic supplementary material:**

The online version of this article (doi:10.1007/s13205-014-0248-3) contains supplementary material, which is available to authorized users.

## Introduction

*Staphylococcus aureus* mostly derives energy from glucose catabolism through glycolysis and Krebs cycles. The end product of glycolysis “Pyruvate” enters into TCA cycle and regulates the energy levels linked to pathogenicity of organism (Venkatesh et al. 2012). Pyruvate kinase (PK) belongs to a group of transferases that couples the free energy of PEP hydrolysis, using K^+^ and Mg^+2^ as co-factors where it generates ATP and pyruvate (Nowak and Suelter 1981). *S. aureus* generates two molecules of pyruvate for every molecule of glucose consumption that ultimately reduces two molecules of NAD+ to NADH creating redox imbalance which facilitates biofilm formation (Shimizu 2014; Ravcheev et al. 2012; Zhu et al. 2007).

“PK” is one of the three regulatory enzymes in glycolysis; it exhibited homotropic positive co-operativity for PEP, but not for ADP. It controls the entire glycolytic pathway by regulating the flux from Fructose-1, 6-bis-phosphate (FBP) to pyruvate (Muñoz and Ponce 2003). The most common form of allosteric regulation for PK is its upregulation by FBP, which increases the affinity and reduces the co-operativity of substrate binding which also depends on bound divalent cations in the active site. Here bound substrate and metal ions increases affinity of FBP for the allosteric site (Bond et al. 2000; Zoraghi et al. 2010, 2011a, b; Kumar et al. 2014). ATP, Alanine, and phenylalanine become negative allosteric inhibitors for PK and serves as a switch between the glycolytic and gluconeogenic pathways. This regulation flux by PK directly affects the concentrations of glycolytic intermediates, biosynthetic precursors, and nucleoside tri-phosphates in the cell. Thus PK controls consumption of metabolic carbon for biosynthesis and utilization of pyruvate for energy production (Shimizu 2014). Therefore; it appears that pyruvate levels are vital in the organism for the biofilm formation and maintaining of reductive conditions (Cramton et al. 1999; Gotz 2002; Yeswanth et al. 2013). In view of importance of pyruvate in this pathogen it was predicted that pyruvate kinase could be potential drug target and various studies using alkaloids as PK inhibitors were proposed as effective drugs against *S. aureus* infections (Zoraghi et al. 2010, 2011a, b); however, the essential role of pyruvate in the biofilm formation continues to pose questions against the efficacy of such compounds in vivo conditions.

The expression of enzymes involved in the cell wall biosynthesis, virulence factors, production of toxins, and purine biosynthesis for both energy production and growth are controlled through phosphorylation by PknB (Beltramini et al. 2009; De´barbouille et al. 2009; Donat et al. 2009; Tamber et al. 2010; Miller et al. 2010). The gene sequence of PK (JN645815) showed the presence of PknB site; therefore, like in *Bacillus anthracis* where fall in PK activity was observed on phosphorylation with PknB (Arora et al. 2012), we predicted that probably phosphorylation of PK might be controlling its function in *S. aureus;* hence the present study is aimed at understanding the effect of PK function on phosphorylation by PknB.

## Materials and methods

In the present study chemicals were obtained from Sisco Research Laboratories Pvt. Ltd., India, Hi-Media Laboratories Pvt. Ltd., India, Sigma-Aldrich, USA, New England Biolabs, USA, and QIAGEN Inc., Valencia, CA, USA.

### Bacterial strains and conditions

*Staphylococcus aureus* ATCC 12600 and *Escherichia coli* DH5α were obtained from Bangalore Genei Pvt Ltd. *S. aureus* was grown on modified Baird Parker agar at 37 °C. After overnight incubation, a single black shiny colony with distinct zone was picked and inoculated in Brain heart infusion (BHI) broth and incubated at 37° overnight. Thus grown *S. aureus* ATCC 12600 culture was used for the isolation of chromosomal DNA (Hari Prasad et al. 2012).

### Pyruvate kinase enzyme assay

The pyruvate kinase enzyme assay was performed using crude and pure pyk (Venkatesh et al. 2012) and the kinetic parameters *V*_max_, *K*_M_, and *K*_cat_ were calculated from Hanes-Woolf plot [S] vs ([S]/V). In all the experiments protein concentration was determined by the method of Bradford (1976).

### Serine/Threonine protein kinase (PknB) assay

PknB activity was determined at 30 °C using novel nonradiolabeled protein kinase spectrophotometric assay with synthetic peptide acting as substrate on a Cyber lab spectrophotometer, USA. PknB assay mixture contained 0.1 M Tris–HCl pH 7.5, 0.1MATP, and 11.8 μM (30 µg/μl) peptide (stpks = NLCNIPCSALLSSDITASVNCAK). 1 µg/µl enzyme fraction (pure His tag PknB) was mixed and incubated at 30 °C for 10 min. The phosphorylated peptide was purified by passing it through Sephadex G-25 column (1 cm × 15 cm); the fractions were eluted with 0.1 M Tris–HCl pH 7.5 and 150 mM NaCl. The enzyme fraction appeared in the void volume, and in elution volume the phosphorylated peptide was obtained. The phosphate covalently bound to the proteins was estimated by adding freshly prepared reagent A (3.4 mM of Ammonium Molybdate dissolved in 0.5 mM H_2_SO_4_, 10 % SDS, 0.6 M l-Ascorbic acid mixed in 6:1:1(v/v/v) ratio) and incubated at 30 °C for 15 min and the absorbance was recorded at 820 nm against blank (0.1 M Tris–HCl pH 7.5 and 150 mM NaCl and reagent A (Clore et al. 2000). The enzyme activity was measured as the amount of phosphorous added per microgram peptide at 30 °C per minute per ml. For this, the calibration curve was developed using standard KH_2_PO_4_ for the estimation of inorganic phosphate and free phosphate was determined by adding reagent A (Fiske and Subbarow 1925). The phosphorylated peptide was further demonstrated by fractionating the eluted peptide on 15 % SDS-PAGE and staining the gel with reagent A; the bluish green-colored band that appeared in the gel indicated that peptide was phosphorylated by the enzyme fraction. Similarly, the auto-phosphorylation property of PknB was also determined and for this the reaction mixture composition was same except in that peptide was not added. The enzyme activity was measured as the amount of phosphorous added per microgram enzyme at 30 °C per minute per ml. Substrate level phosphorylation was performed by taking different substrate concentrations of 10–120 µM of synthetic peptide, keeping the ATP concentration constant, and the corresponding velocities were calculated and a graph of [S] vs [S/V] (Hanes-Woolf) was plotted, from the graph *K*_M_ and *V*_max_ was determined. For auto phosphorylation activity of PknB the same enzyme assay was carried out except in that peptide was not added. Similarly, the Km, *V*_max_ for auto phosphorylation of PknB was determined by Hanes-Woolf plot.

### Cloning of PknB and PK genes

PknB and PK genes were PCR amplified from chromosomal DNA of *S. aureus* ATCC 12600 and sequenced (Table 1); the amplified products were purified with NP-PCR Purification kit, Taurus Scientific, USA, and sequenced by dye terminating method at MWG Biotech India Ltd, Bangalore, India, and Xcelris Pvt. Ltd. Ahemadabad, India. Thus obtained gene sequences were deposited at GenBank (www.ncbi.nlm.nih.gov/genbank/submt.html) (Table 1) (Ohta et al. 2004). The PCR products were made into proper blunt ends using Klenow fragment (New England Bio labs, USA) and cloned in the Sma I site of pQE30 vector and transformed into *E. coli* DH5α, and generated clones were named as PV 1 and PK 1. The genes in clones PV 1 and PK 1 were over expressed with 0.75 mM IPTG and 1 mM IPTG for 2 and 5 h, respectively. The pure enzymes were obtained by passing the cytosolic fraction of each clone through nickel metal chelate column (Prasad et al. 2013). The expressed proteins were fractionated on 10 % SDS-PAGE and transferred on to nitrocellulose membrane (NCM) (Towbin et al.^1979^). The NCM was blocked with 2 % gelatin and treated with antiHis-tag monoclonal antibody kit (Qiagen) following the manufacturer’s method.

**Table 1:** PCR amplification and sequencing of PknB and PK genes of *S. aureus* ATCC 12600

Gene	Primers	Conditions	Product size	Gene accession numbers
*PknB*	FP: 5′-CATGATAGGTAAAATA-3′ RP: 5′-TTATACATCATCATA-3′	Initial denaturation: 94 °C for 10 min. 40 cycles of denaturation: 94 °C for 60 s annealing: 29.2 °C for 30 s. amplification: 72 °C for 120 s and final elongation: 72 °C for 10 min	2 kb	JN695616
*PK*	FP: 5′-CGACCAGCTTCAGAATC-3′ RP: 5′-GAGCAGCATCAATCGT-3′	Initial denaturation for 10 min at 94 °C; 40 cycles of each having denaturation at 94 °C for 60 s, annealing at 43 °C for 35 s and amplification at 72 °C for 120 s final extension step at 72 °C for 5 min	1.6 kb	JN645815

Multiple sequence alignments were carried out to understand the sequence similarities and dissimilarities. The *PK* sequences were retrieved for *Staphylococcus aureus*, *Escherichia coli*, *Pyrobaculum aerophilum, Aeropyrum pernix, Streptococcus mutans, Bacillus licheniformis, Salmonella typhimurium, and Homo sapiens R/L isoform* from GenBank. All these structures were subjected to CLUSTAL W software and the results were recorded (Thompson et al. 1997).

### In vitro regulation of PK

PK was phosphorylated with PknB; the assay mixture contained 0.1 M Tris–HCl pH 7.5, 0.1 M ATP, 1 µg/ml pure enzyme (PK), and 3 µg/ml pure PknB was mixed and incubated at 30 °C for 10 min. The phosphorylated enzyme (PK) was purified by passing through Sephadex G-25 column (1 cm × 15 cm); the fractions were eluted with 0.1 M Tris–HCl pH 7.5 and 150 mM NaCl. The enzyme fractions appeared in the void volume. The bound phosphorous was estimated by adding freshly prepared reagent A and incubated at 30 °C for 15 min, and the absorbance was recorded at 820 nm against blank (0.1 M Tris–HCl pH 7.5 and 150 mM NaCl and reagent A). The phosphorylated enzymes were used to carry out the enzyme assay as described earlier. These phosphorylated proteins were fractionated in 10 % SDS-PAGE and the bound phosphate was detected by immersing the gel in reagent A and followed by staining with Coomassie Brilliant blue R_250_.

In all the experiments protein concentration was determined by the method of Bradford (1976).

## Results

### Cloning, expression, and characterization of *PK* gene

**Fig. 1:**
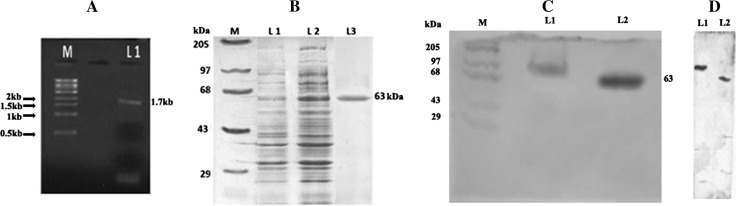
**a** Electrophoretogram showing PCR amplification of pyruvate kinase from the chromosomal DNA of *S. aureus* ATCC 12600. *Lane M* super mix marker obtained from Merek Biosciences Pvt Ltd. *L1* Amplified 1.7 kb PCR product. **b** SDS-PAGE analysis of analysis pyruvate kinase: electrophoretogram showing the pyruvate kinase protein obtained from recombinant clone PK 1. *Lane M* High-molecular-weight marker from Merek Biosciences Pvt Ltd. *Lane 1* Cytosolic fraction of clone PK 1. *Lane 2* Cytosolic fraction of clone PK 1 induced with IPTG. *Lane 3* Purified PK obtained by passing the cytosolic fraction of IPTG-induced PK 1 clone through Nickel metal chelate agarose column. **c** In vitro phosphorylation assay: SDS-PAGE gel was first stained with reagent A, followed by Coomasie brilliant blue R250 staining. *Lane M* High-molecular-weight marker from Merek Biosciences Pvt Ltd. *Lane L1* phosphorylated PK obtained from Sephadex G-25 column. *Lane L2* Pure PK obtained from Nickel metal chelate agarose column. **d** Western blot using an anti-His tag antibody: *Lane L1* phosphorylated PK obtained from Sephadex G-25 column. *Lane L2* Pure PK obtained from Nickel metal chelate agarose column

**Fig. 2:**
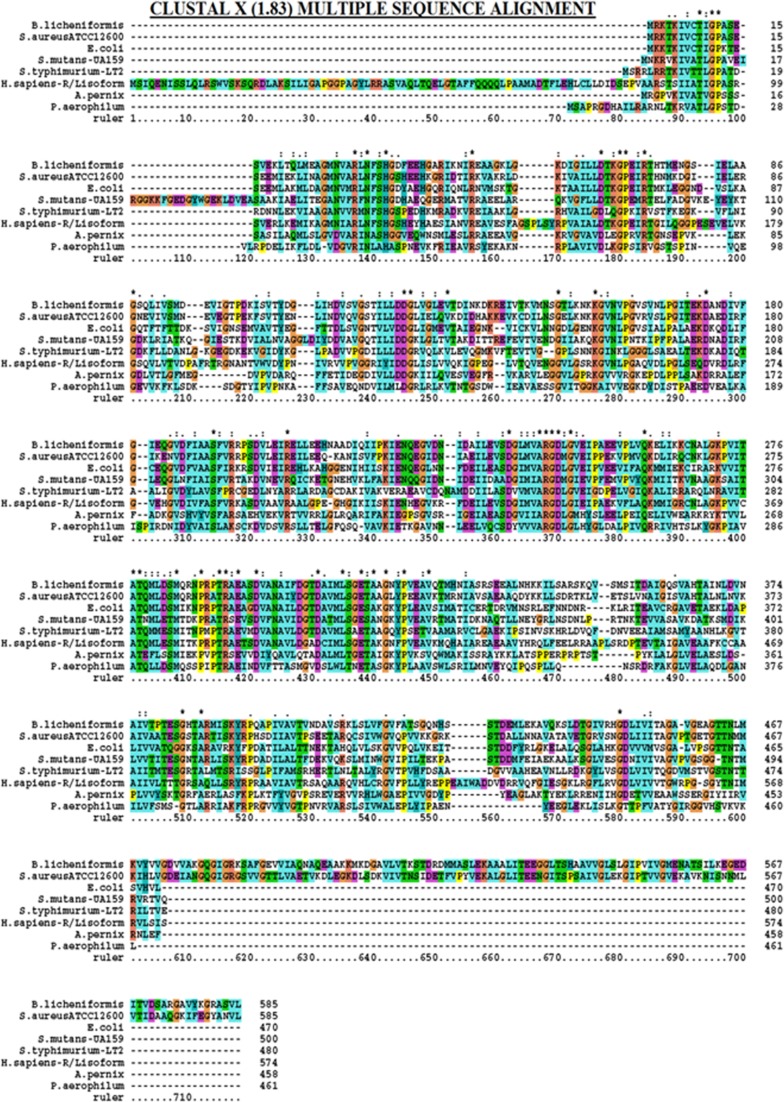
Multiple sequence alignment of Pyruvate kinase: Amino acid sequences of Pyruvate kinase from various organisms were compared with the amino acid sequence of Human pyruvate kinase using CLUSTAL X

In the present study, the PK gene (1.7 kb) was PCR amplified from the chromosomal DNA of *S. aureus* ATCC 12600 and cloned in the Sma I site of pQE30 vector (Fig. 1a) in −1 frame, and the clone was named as PK 1. In order to ascertain the PK gene was sequenced (Supplementary Fig. 1) using the same primers and after ensuring the correct sequence (Accession number: JN645815), the enzyme was expressed in *E. coli* DH5α with 1 mM IPTG; thus expressed PK gene was purified by passing through nickel metal chelate agarose column. The pure recombinant PK exhibited single band in SDS-PAGE with a molecular weight of 63 kDa corresponding to the monomeric form of the enzyme (Fig. 1b) and the expression was validated using anti-His tag antibody (Fig. 1d). The PK sequence showed complete homology with all the PK gene sequence reported for other strains of *S. aureus,* indicating the presence of only one PK in this pathogen (Fig. 2). The rPK kinetics was close to the native PK (Table 2). The formation of pyruvate was higher in *S. aureus* grown in BHI broth compared to LB broth (Table 3).

**Table 2:** Enzyme kinetics of PK

Source of pyruvate kinase (cytosolic fraction)	*V*_max_ (μMNADH/mg/min)	*K*_m_ (μM)	*K*_cat_ (min^−1^)
Native PK in the cytosolic fraction of *S. aureus* ATCC 12600	57.1 ± 0.24	0.7 ± 0.05	157.5
r PK 1	76.92 ± 0.82	0.69 ± 0.02	158.84

Values are the mean ± SD obtained from three determinations

**Table 3:** Pyruvate kinase enzyme kinetics in the cytosolic fractions of *S. aureus* ATCC 12600 grown in LB and BHI broths

LB broth			BHI broth		
*K*_m_ (µM)	*V*_max_ (µM/mg/min)	*K*_cat_ (min^−1^)	*K*_m_ (µM)	*V*_max_ (µM/mg/min)	*K*_cat_ (min^−1^)
0.74 ± 0.05	0.092 ± 0.12	46	0.75 + 0.05	57.1 ± 0.24	157.5

Values are the mean ± SD obtained from three determinations

On scanning the annotated protein sequence of *S. aureus* PK in PROSITE (Altschul et al. 1997) the following exclusive sites were observed: casein kinase phosphorylation, *N*-myristoylation, *N*-glycosylation, protein kinase C phosphorylation, cAMP and cGMP-dependent protein kinase phosphorylation, cell attachment sequence, and Amidation sites which all indicate the pyk of *S. aureus* is a unique enzyme. The Multiple sequence alignment results showed very low amino acid sequence identity with other bacterial PK and human pyk (Fig. 2).

### Cloning, expression, and characterization of PknB gene

The gene encoding PknB (2.0 kb) was amplified from *S. aureus* ATCC 12600 chromosomal DNA and sequenced; the sequence (JN695616) showed complete homology with PknB of several *S. aureus* strains in the reported databases. The sequence analysis of PknB enzyme showed the presence of catalytic domain between 10th and 267th residues which contains 12 specific Hank motifs, both ATP and substrate binding sites similar to eukaryotic protein kinases. Transmembrane domain followed by three different duplicated forms of PASTA domains. PASTA1 distributed between 377th and 440th residues, PASTA2 distributed between 445th and 508th residues, and PASTA3 distributed between 514th and 577th residues in the annotated protein sequence of PknB gene and between these two domains is a single transmembrane segment. These unique characters are the features exhibited by several PknB enzymes expressed in different strains of *S. aureus*.

**Fig. 3:**
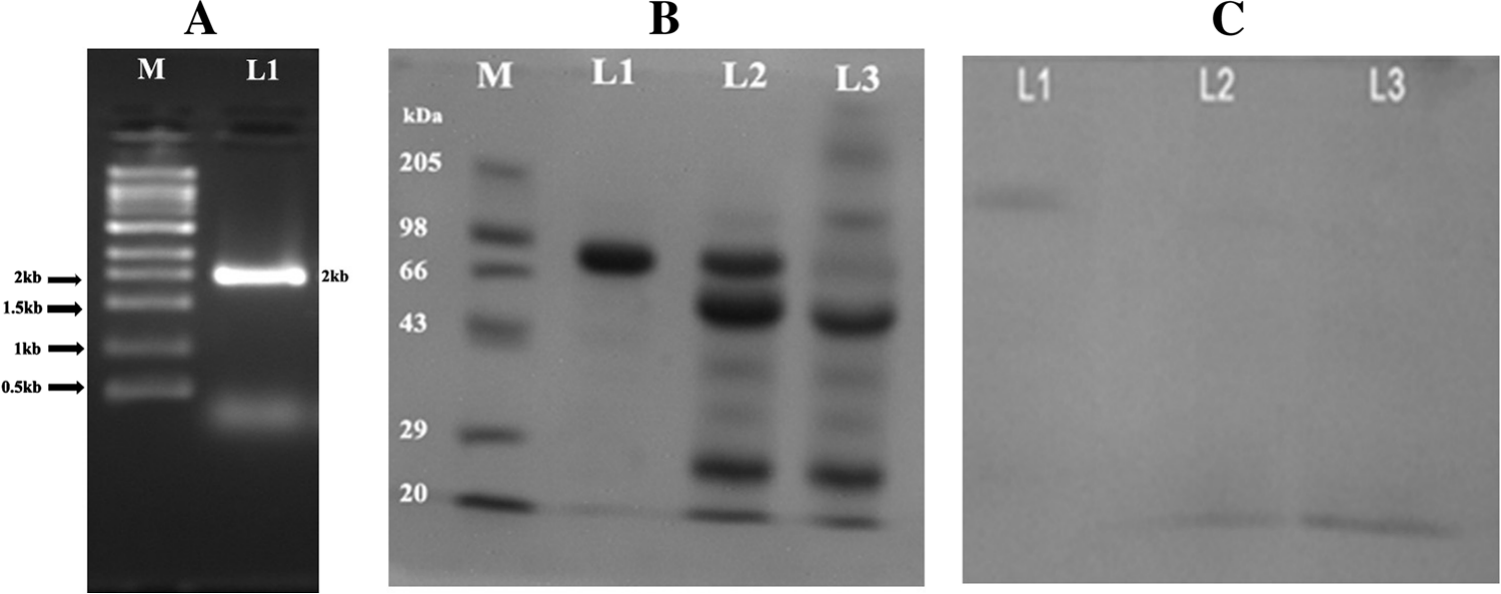
**a** Electrophoretogram showing PCR amplification of PknB from the chromosomal DNA of *S. aureus* ATCC 12600. *Lane M* super mix marker obtained from Merek Biosciences Pvt Ltd. *L1* Amplified 2 kb PCR product. **b** SDS-PAGE analysis of analysis PknB: electrophoretogram showing the PknB protein obtained from recombinant clone PV 1. *Lane M* High-molecular-weight marker from Merek Biosciences Pvt Ltd. *Lane L1* Purified PknB obtained by passing the cytosolic fraction of IPTG-induced PV 1 clone through Nickel metal chelate agarose column. *Lane L2* Cytosolic fraction of clone PV 1 induced with IPTG. *Lane L3* Cytosolic fraction of clone PV 1. **c** In vitro phosphorylation assay: SDS-PAGE gel stained with reagent A. *Lane L1* phosphorylated pure PknB obtained from Sephadex G-25 column. *Lane L2* and *L3* phosphorylated substrates of stpks obtained from Sephadex G-25 column

The PknB gene was cloned in pQE 30vector and the clone was called as PV 1. PknB gene in this clone was expressed with 0.75 mM IPTG which resulted in the successful expression of the gene and the enzyme was purified by passing through nickel metal agarose column. The molecular weight of the rPknB was found to be 73 kDa which corresponds to the insert cloned and is equivalent to the monomeric form of Pkn B protein (Fig. 3a, b). The kinetics of PknB is explained in Table 4. The rPknB exhibited both substrate level phosphorylation and autophosphorylation; the phosphorylated molecules were separated on SDS-PAGE, and the appearance of blue colored bands on staining with reagent A confirms the presence of phosphate in the enzyme and substrate stpks (Fig. 3c).

**Table 4:** Enzyme Kinetics of rPknB

Source of enzyme	Protein enzyme concentration (µg/µl)	Enzyme activity (µM/ml/min)	*K*_m_ (mM)	*V*_max_ (µM/mg/min)
PknB (substrate level)	4	0.162 ± 0.08	0.720 ± 0.08	1.98 ± 0.2
r PknB (auto phosphorylation)	4	0.316 ± 0.090	0.380 ± 0.07	8.0 ± 0.43

Values are the mean ± SD from three determinations

### In vitro phosphorylation of PK

The presence of PknB site in the PK gene sequence encouraged us to carry out phosphorylation of PK. On phosphorylation with PknB, the bound phosphate was identified by its ability to react with reagent A which was identified spectrophotometrically. On fractionating these phosphorylated enzymes in 10 % SDS-PAGE and immersing the gel in reagent A, emergence of blue colored bands indicated PK was phosphorylated (Fig. 1c). The mobility of phosphorylated PK was higher than the native pure PK (Fig. 1c). The phosphorylated PK (P-PK) exhibited reduced activity (40 %) compared to the native PK (PK = 0.2 ± 0.015 μM NADH/min/ml to P-PK = 0.12 ± 0.01 μMNADH/min/ml) (Table 5).

**Table 5:** PK Regulation by phosphorylation and dephosphorylation

PK	Enzyme activity units (µMNADH/ml/min)
Pure rPK	0.2 ± 0.015
PK-P by PknB	0.12 ± 0.01

Values are the mean ± SD from three determinations

## Discussion

*Staphylococcus aureus* is a death-defying pathogen of both animals and humans that can cause minor skin infections to major life-threatening diseases. Phosphorylation of host proteins due to the secretary PknB has been implicated its ability to grow in any anatomical organs of human host (Lowy 1998; Miller et al. 2010). The expression of PknB in *S. aureus* is involved in controlling metabolic stress and regulation of plethora of metabolic pathways accordingly; we have found 40 % decreased PK activity on phosphorylation with PknB (Table 5). This response is upregulated in anaerobic growth conditions where Redox-sensing repressor Rex is reported to be involved in the regulation of anaerobic respiration in response to the NADH/NAD^+^ levels; in these conditions the association between pyruvate and TCA cycle is reported to be very weak, thus leading to more biosynthesis of toxins, virulence factors, and PIA synthesis which are highly favored for biofilm formation (Cramton et al. 1999; Beltramini et al. 2009; De´barbouille et al. 2009; Donat et al. 2009; Tamber et al. 2010; Liu et al. 2011; Strasters and Winkler 1963; Zhu et al. 2007; Pagels et al. 2010; Ravcheev et al. 2012).

In *S. aureus*, shift of growth conditions from aerobic to anaerobic increased the expression of glycolytic enzymes, such as GapA, Eno, Pgk, and Pyk (Fuchs et al. 2007); in our previous studies we have also shown elevated biofilm units and lactate dehydrogenase activity when *S. aureus* was grown in BHI broth with increased concentrations of glucose (Yeswanth et al. 2013). This enhanced glycolysis suppresses Krebs cycle resulting in the accumulation of lactate, acetate, formate, and acetoin, suggesting that glucose is catabolized to pyruvate further, catabolization via the lactate dehydrogenase, pyruvate formate-lyase, and butanediol pathway leading to biofilm formation (Zhu et al. 2007). In congruence with these observations in the present study, we have observed elevated PK activity in *S. aureus* grown in BHI broth compared to LB broth (Table 3). All these results conclusively explain the pyruvate formation in anaerobic condition favors more synthesis than energy generation contributing to the formation of biofilms which is one of the key pathogenic factors.

## Conclusion

*Staphylococcus aureus* has unique feature to colonize on any anatomical locales of human body. This character makes the organism to spread its pathogenesis at a rapid rate. In this context, we found that PK catalyzes the irreversible conversion of Phosphoenolpyruvate to pyruvate that regulates the metabolic flux which is controlled by the expression of PknB. This PknB regulates functioning of PK thus controlling the levels of pyruvate in the organism. Therefore, high pyruvate formation in anaerobic conditions does not contribute to energy generation, but favors upregulating biosynthetic pathways involved in biofilm formation which is one of the key pathogenic factors.

## Electronic supplementary material

Below is the link to the electronic supplementary material.

Supplementary material 1 (PDF 214 kb)
