# Supplementary figures and images for: In Staphylococcus aureus the regulation of pyruvate kinase activity by serine/threonine protein kinase favors biofilm formation
Source: 3 Biotech. 2014 Sep 12;5(4):505–12. doi: 10.1007/s13205-014-0248-3 (PMC4522715; doi:10.1007/s13205-014-0248-3)

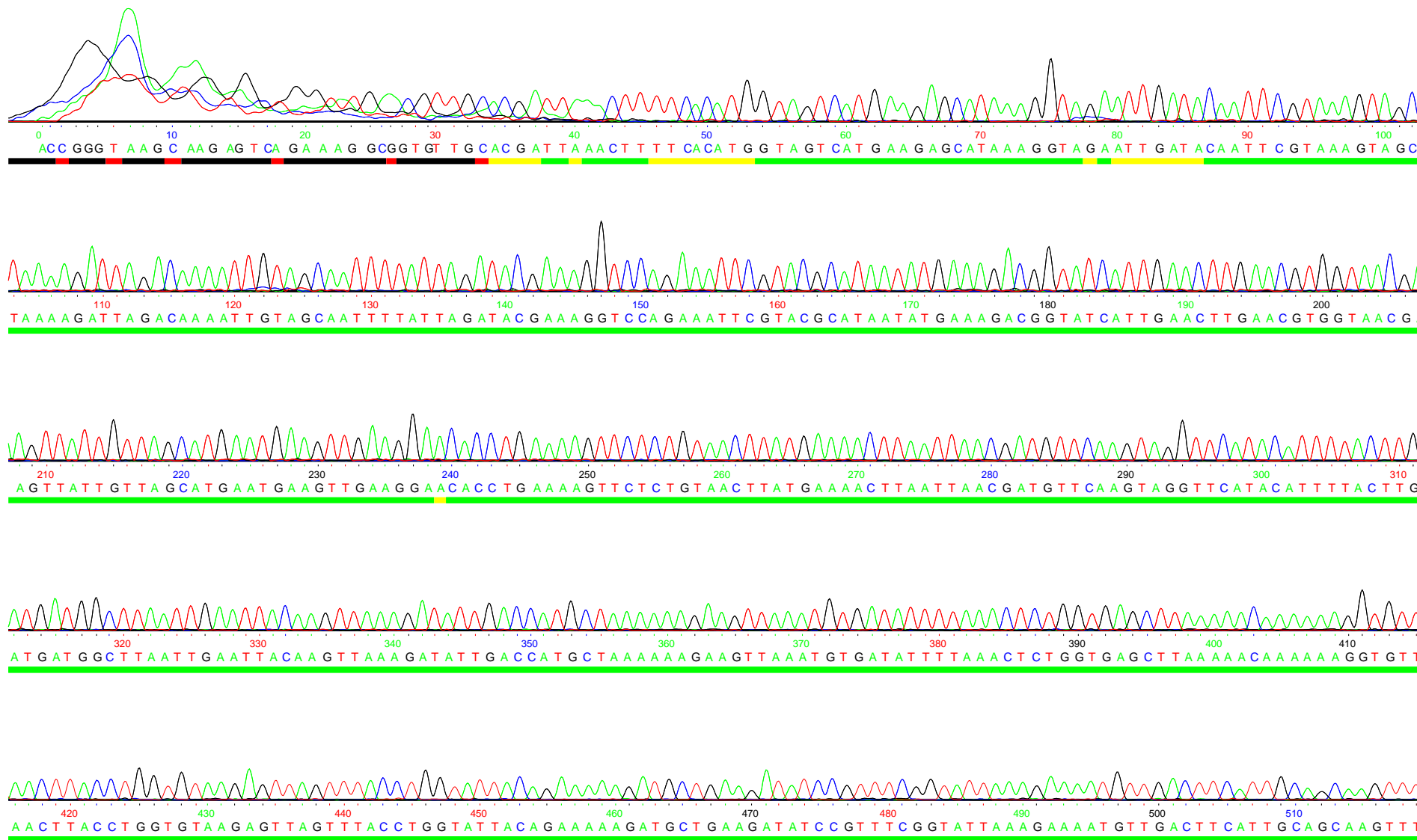

Quality colorcoding

0-9

10-19

20-29

>30

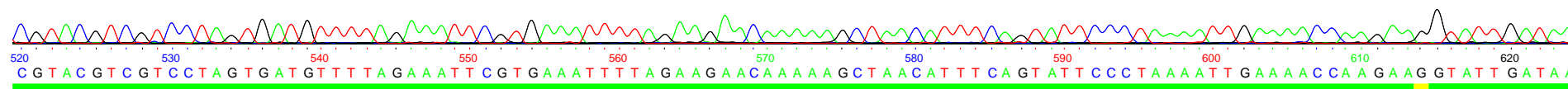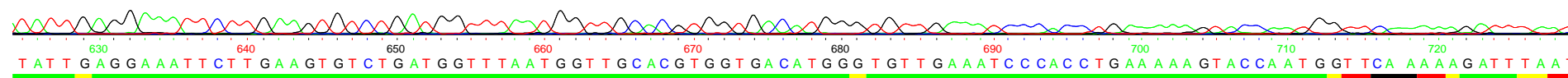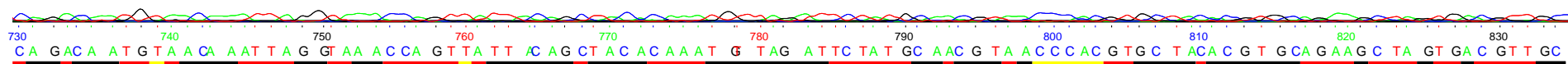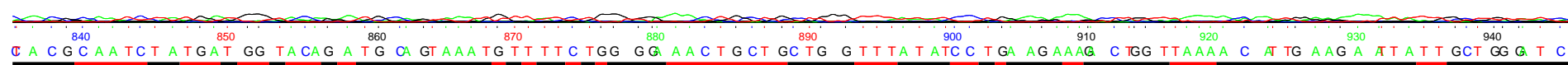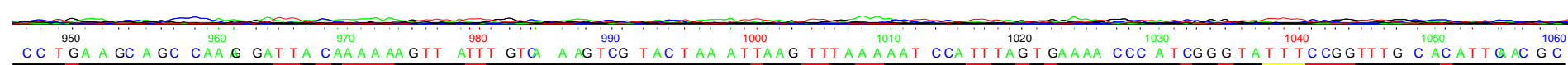

Quality colorcoding

0-9

10-19

20-29

>30

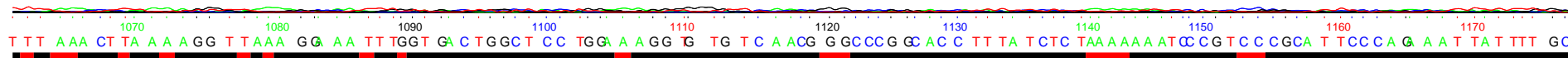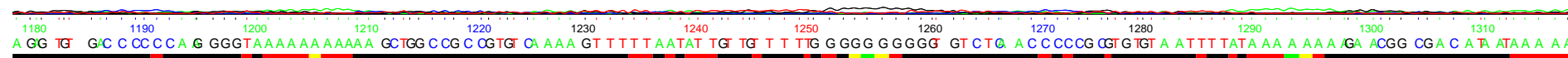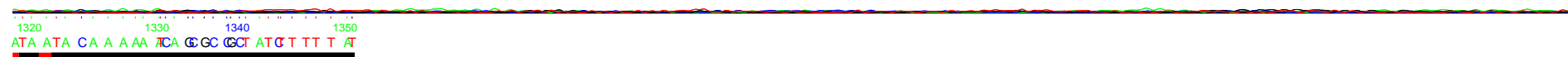

Quality colorcoding

0-9

10-19

20-29

>30

Supplement: Supplementary file 1 — Supplementary material 1 (PDF 214 kb) [file 13205_2014_248_MOESM1_ESM.pdf]
